# A side-by-side comparison of the new VITEK MS PRIME and the MALDI Biotyper sirius in the clinical microbiology laboratory

**DOI:** 10.1007/s10096-023-04666-x

**Published:** 2023-10-05

**Authors:** Philipp Thelen, Sandra Graeber, Erika Schmidt, Axel Hamprecht

**Affiliations:** 1 Institute of Medical Microbiology and Virology, Carl von Ossietzky University of Oldenburg, Oldenburg, Germany; 2 Institute for Medical Microbiology and Virology, Klinikum Oldenburg, Oldenburg, Germany

**Keywords:** MALDI-TOF MS, Laboratory management, Rapid identification, VITEK MS PRIME, MALDI Biotyper sirius

## Abstract

**Purpose:**

This study aims to evaluate the performance of two latest generation matrix-assisted laser desorption ionization-time of flight mass spectrometry (MALDI-TOF MS) systems in routine laboratory settings, focusing on turnaround time (TAT), time to results (TTR), hands-on time, and identification rate.

**Methods:**

We conducted a time and motion study on three workflow scenarios to simulate different laboratory settings. Overall, 618 bacterial isolates from a tertiary hospital’s laboratory were processed using the VITEK MS PRIME (bioMérieux) and the MALDI Biotyper sirius (Bruker Daltonics) and their corresponding databases VITEK IVD Database 3.2 and MBT reference library 12.

**Results:**

The target preparation process showed no significant difference in TAT, but the Biotyper workflow had a shorter hands-on time by 3 to 6 min. In the measurement process, TTR was three to five times shorter for the Biotyper sirius while hands-on time was significantly shorter for VITEK MS PRIME (approximately 1.5 min per target). The identification rate without retesting was 97.9% for VITEK MS PRIME and 98.9% for Biotyper sirius. Both systems achieved 100% agreement at genus and 96.2% at species level.

**Conclusion:**

Both systems exhibited excellent identification rates for routine bacterial isolates. Due to its high speed, the Biotyper sirius is suited for laboratories with high sample throughput and a workflow designed for processing larger batches. The VITEK MS PRIME, with its “load and go” system accommodating up to 16 targets, reduces hands-on time, making it a reasonable choice for laboratories with fewer identifications overall but a higher number of targets and a workflow designed for parallel processing on different workstations.

**Supplementary Information:**

The online version contains supplementary material available at 10.1007/s10096-023-04666-x.

## Introduction

Matrix-assisted laser desorption ionization-time of flight mass spectrometry (MALDI-TOF MS) has become the gold standard for the routine identification of bacteria and yeasts in clinical microbiology laboratories (CML) since the first successful application of the technology for microbial identification in 1996 [1, 2] and introduction of commercial IVD certified systems a decade ago [3]. Compared to classic biochemical identification, MALDI-TOF MS is rapid, cost-effective, and accurate. Optimized sample preparation and constantly updated databases today allow the identification of rare bacterial species as well as mycobacteria and filamentous fungi [4]. In recent years, the use of MALDI-TOF MS expanded to the rapid detection of antibiotic resistance [5].

At the same time, shortage of specialized staff has been an ongoing challenge for medical laboratories in the USA [6, 7], UK, and Europe [8–10] and was aggravated by the COVID-19 pandemic. Optimization of current workflows, introduction of new technologies, automation, and advanced IT support can help to cut down technician’s hands-on time as one component to meet this challenge.

The decision on new diagnostic systems is a major investment not only from a finance point of view since it also affects laboratory organization.

We therefore compared two latest generation MALDI-TOF MS systems, the VITEK MS PRIME (bioMérieux) and the MALDI Biotyper sirius (Bruker Daltonics), for their performance in different routine laboratory settings. The primary endpoint of the study was technician hands-on time, turnaround time, and time to results for the identification of bacterial isolates. Furthermore, the rate of identification and agreement of both systems were evaluated.

## Materials and methods

### Bacterial isolates

A total of 618 non-duplicate bacterial isolates from the microbiological laboratory at a tertiary care hospital were processed. The isolates originated from different clinical specimens such as urine, blood cultures, and swabs. Target preparation was done using biomass harvested after 16–20 h of incubation at 35 ± 2 °C from different media: CHROMagar Orientation plates (ChromAgar, Paris, France) for non-fastidious gram-negative bacteria (ambient air), BD Columbia agar + 5% sheep blood (BD, Heidelberg, Germany) for non-fastidious gram-positive bacteria, or BD Chocolate agar for fastidious bacteria (both 5% CO_2_ atmosphere). Anaerobic bacteria were processed after 72 h of incubation at 35 ± 2 °C on BD Schaedler agar or BD Schaedler KV agar under anaerobic conditions.

### MALDI-TOF MS systems and software

Two MALDI-TOF MS systems were evaluated, the MALDI Biotyper sirius (Bruker Daltonics, Bremen, Germany) and the VITEK MS PRIME (bioMérieux, Marcy l’Etoile, France). The MS spectra obtained by MALDI Biotyper sirius were analyzed with the MBT Compass HT software (version 5.1.300) and MBT reference library (version 12.0.0.0). The spectra generated by VITEK MS PRIME were analyzed by the VITEK MS Software (version 1.1.0–203571250) and the VITEK MS IVD Database 3.2.

### Target preparation

**Fig. 1:**
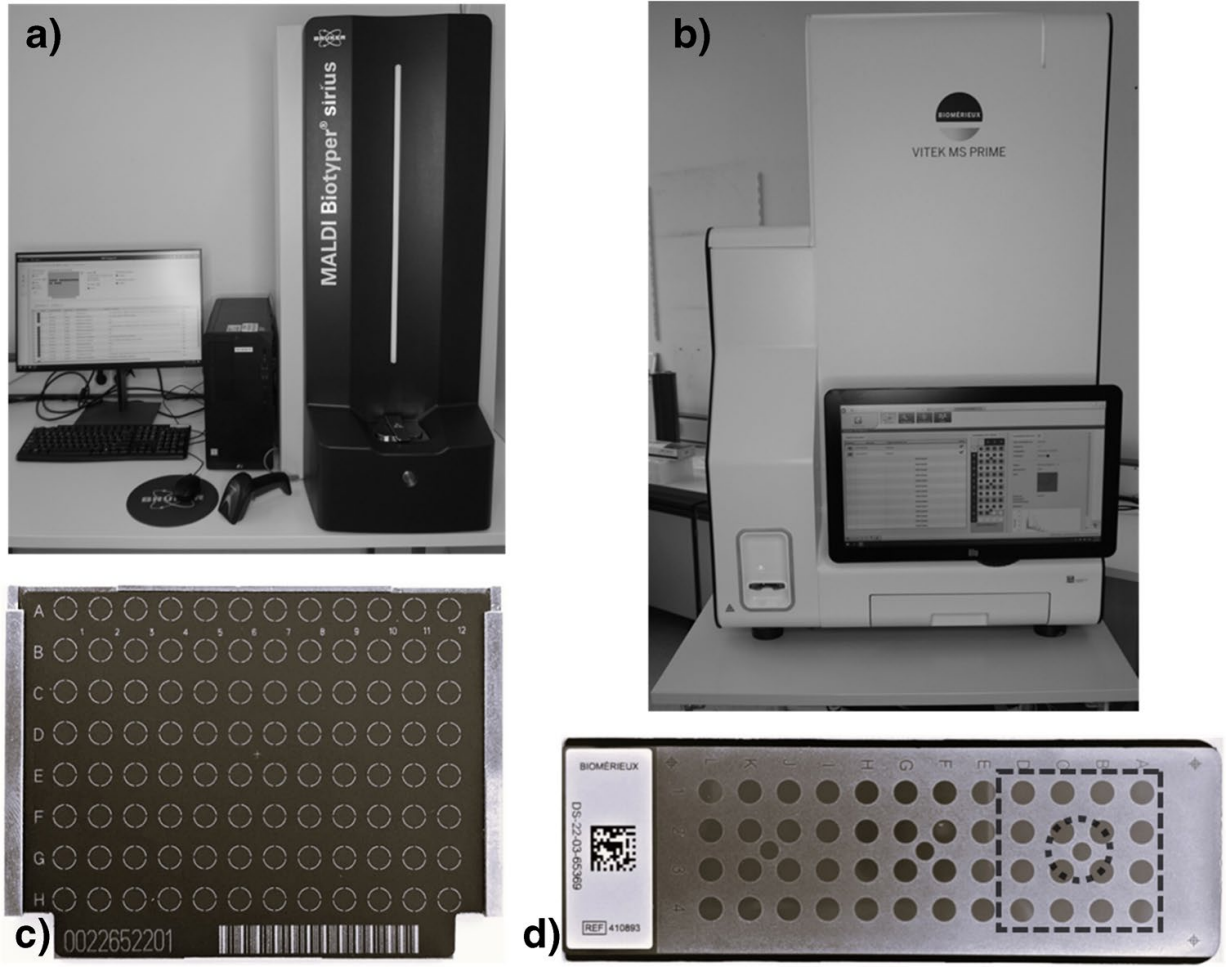
**a** Biotyper sirius setup. **b** VITEK MS PRIME setup. **c** Bruker Biotarget 96 with target carrier. **d** VITEK MS-DS target slide; the dashed square corresponds to one acquisition group of 16 spots; the dotted circle highlights the spots used for calibration

With both systems, single-use targets were used (Fig. 1). The MBT Biotarget 96 has a capacity of 96 spots. For each run, one bacterial test standard (Bruker IVD BTS) was prepared, corresponding to one spot on the target. The VITEK MS-DS target has a capacity of 48 samples and 3 separate spots for calibration with a test standard (*E. coli* ATCC 8739). The slide is split into three acquisition groups with 16 sample spots (dashed square Fig. 1) and three corresponding calibration spots (dotted circle Fig. 1). Each section can be used once regardless of sample size (1–16 spots).

For all bacterial isolates, a single spot was prepared using the direct transfer protocol (DT) as described by the manufacturers. Briefly, biomass from an overnight culture was applied to a target spot using a wooden toothpick for the Biotyper workflow or with a VITEK PICKME PEN for the VITEK MS PRIME workflow. The spot was covered with MALDI Matrix (alpha-cyano-4-hydroxycinnamic acid). For the Biotyper workflow, samples were overlaid with Bruker HCCA matrix within 30 min of application, whereas each sample was covered directly after application with VITEK MS CHCA matrix for the VITEK MS PRIME workflow according to the manufacturer’s IVD guidelines. Matrix was allowed to dry at room temperature for both workflows.

### Time and motion study for different workflow strategies

**Fig. 2:**
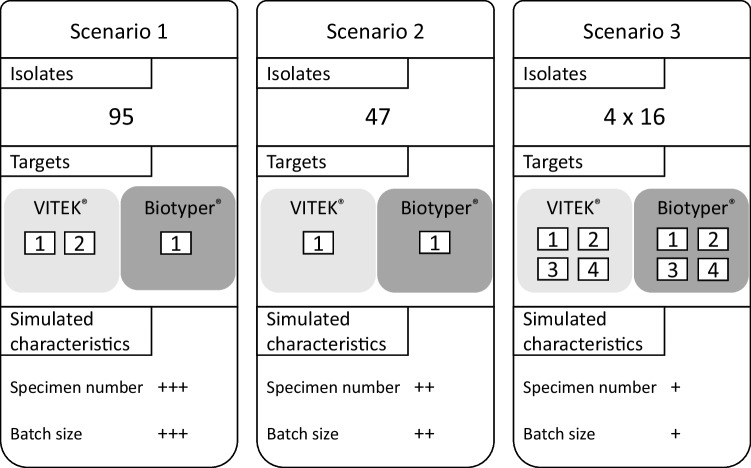
Three scenarios simulated different laboratory characteristics and preferred workflows. +  +  + , high; +  + , moderate; +, low

In order to evaluate workflow performance in different laboratory settings, we performed time and motion studies for three different scenarios (Fig. 2). Each of the three scenarios was operated by three technicians.

In scenario one, 95 isolates were processed on one MALDI Biotyper target and two VITEK MS PRIME targets. In scenario two, 47 isolates were processed on one Biotyper and one VITEK target. In scenario three, four sets of 16 bacterial isolates were processed on four targets, loaded either subsequently after each measurement run to the MALDI Biotyper sirius or semi-automated with the “load and go” system on the VITEK MS PRIME.

The turnaround time (TAT) and hands-on time of the target preparation process and the time to results (TTR) and hands-on time of the measurement process were recorded separately using a stopwatch. Hands-on time is defined as the fraction of the process in which technicians are actively involved. This comprises target preparation, interaction with the software, and loading the target into the MALDI-TOF MS machines.

TAT reflects the time needed for the entire process of target preparation including hands-on time and drying of matrix. The target preparation process begins by creating a new project in the software and ends when the matrix on all spots is completely dried.

The TTR reflects the time needed for the entire MALDI-TOF MS measurement process including hands-on time, automated target handling, and spectra acquisition and analysis. The process begins with loading the target to the machine and ends when the last spectrum of the run is analyzed by the respective software.

### Identification criteria

For both systems, the IVD identification criteria were used as recommended by the manufacturers. Briefly, for the MALDI Biotyper sirius, MS spectra that result in a log(score) of ≥ 2.0 for the similarity to peak patterns in the database are considered high-confidence identifications. If the first and second best match yield different species but belong to the same genus despite log(score) ≥ 2.0, these are considered low-consistency identifications. MS spectra that provide a log(score) of 1.7 to 1.99 are considered low-confidence identifications. A log(score) < 1.7 indicates no identification.

For the VITEK MS PRIME, MS spectra that provide confidence values of 60 to 99.9% for the similarity to a reference species in the database are considered high-confidence identifications. Identifications are defined as low discrimination when a spectrum matches to two, three, or four species equally. No ID is reported when there is no match in the database or when more than four species are matched, which results in a confidence value < 60%.

### Agreement

Agreement of identification on species and genus level of both systems was calculated including high- and low-confidence identifications. If agreement could not be determined due to non-identification with one system, isolates were retested.

### Statistical analysis

The two-sided paired *t* test was used for statistical analysis of the duration of TAT, TTR, and hands-on time. Differences in identification rate were analyzed by chi-square test. Findings were considered significant if the *p* value was < 0.05.

### Usability

Aspects of usability were included in our comparison. These aspects included properties of the machines, such as dimensions, noise emission, capacity per target, and loading capacity per machine. Furthermore, practical considerations like the ease of target preparation, calibration procedure, and matrix preparation were compared based on technicians´ experience during the study.

## Results

### Target preparation

Overall turnaround time (TAT) for the target preparation process including drying of matrix did not differ significantly between both systems. TAT was ~ 40, ~ 24, and ~ 30 min for the preparation of 95 (scenario 1), 47 (scenario 2), and 4 × 16 (scenario 3) isolates for both systems (Table 1). Regarding technician hands-on time, the Biotyper sirius workflow was 6.3 min (95% CI − 0.9 to 13.6), 3.6 min (95% CI 0.7 to 6.5), and 2.7 min (95% CI 2.2 to 3.2) faster when processing 95, 47, and 4 times 16 isolates, respectively.

**Table 1:** Mean duration of turnaround time (TAT) and hands-on time for the target preparation process and mean time to results (TTR) and hands-on time for the MALDI TOF measurement process (*n* = 3)

	Biotyper sirius	VITEK MS PRIME	Mean difference	95% CI	*P*-value
Target preparation					
Scenario **1** (95 isolates)					
TAT (min)	38.1	40.4	2.3	–3.3 − 7.9	0.22
Hands-on time (min)	29.4	35.8	6.3	–0.9 − 13.6	0.06
Scenario **2** (47 isolates)					
TAT (min)	23.7	24.4	0.7	–0.8 − 2.2	0.17
Hands-on time (min)	15.8	19.4	3.6	0.7 − 6.5	< 0.05
Scenario **3** (4×16 isolates)					
TAT (min)	29.2	30.1	0.9	–2.2 − 4.0	0.33
Hands-on time (min)	22.1	24.8	2.7	2.2 − 3.2	< 0.05
MALDI-TOF MS measurement					
Scenario **1** (95 isolates)					
TTR (min)	9.0	48.2	39.4	37.6 − 41.3	< 0.05
Hands-on time (min)	2.5	0.6	1.9	0.6 − 3.2	< 0.05
Scenario **2** (47 isolates)					
TTR (min)	5.7	23.7	18.0	16.6 − 19.3	< 0.05
Hands-on time (min)	1.8	0.3	1.5	1.1 − 1.9	< 0.05
Scenario **3** (4×16 isolates)					
TTR (min)	14.4	43.5	29.5	27.6 − 30.7	< 0.05
Hands-on time (min)	7.8	1.1	6.7	5.9 − 7.5	< 0.05

### MALDI-TOF MS measurement

In the MALDI-TOF MS measurement process, significant differences between both systems were observed (Table 1). The time to results (TTR) in scenarios 1, 2, and 3 was 39.4, 18.0, and 29.5 min shorter for the Biotyper sirius compared to the VITEK MS PRIME, respectively. For scenario 1, simulating a high-throughput laboratory with a workflow optimized for large batches, the Biotyper workflow is about five times faster due to its high measurement speed. By contrast, in scenario 3, which resembles a laboratory with fewer numbers of identifications but higher number of targets, the TTR for the Biotyper sirius was approximately three times faster. This difference between the scenarios is caused by the significantly shorter hands-on time with the VITEK MS PRIME. In scenario 3, where four targets were loaded, hands-on time was 6.7 min shorter for the VITEK MS PRIME workflow.

### Identification

Both systems achieved high rates of identification (98.9% for Biotyper sirius and 97.9% for VITEK MS PRIME) and agreement both at the genus (100%) and the species (96.3%) level in the routine laboratory setting with just a single attempt per isolate (Table 2). Identified species were mostly *Enterobacterales* followed by *Staphylococcus* spp*.* and *Enterococcus* spp*.* The distribution of species and summary of test scores is illustrated in the Online Resource Fig. 1 and Table 1.

**Table 2:** Rate of identification and agreement of both MALDI-TOF MS systems after single measurement

	Biotyper sirius	VITEK MS PRIME	
	*N* (%)	*N* (%)	*P* value
Total isolates	618	618	
Isolates with ID	611 (98.9)	605 (97.9)	0.18
No ID	6 (1.0)	10 (1.6)	0.31
No spectrum aquired	1 (0.1)	3 (0.5)	0.31
Score ≥ 2.0 (high consistency)	587 (95.0)		
Score ≥ 2.0 (low consistency)	7 (1.1)		
Score 1.7–1.99	17 (2.8)		
Confidence value 99.9%		568 (91.9)	
Confidence value 60–99.8%		11 (1.8)	
Low discrimination		26 (4.2)	
Pair of ID	598		
Agreement on genus level	598 (100.0)		
Agreement on species level	576 (96.3)		

There was no bacterial isolate that could not be identified by both systems. Of the 20 isolates that were not identified on the first attempt of testing by either system, 14 were tested again with the same protocol used for initial identification. The other six isolates were lost in the follow-up and were excluded from further analysis as they were not retained after the first measurement. All retested isolates were identified in the retesting run. Including the retesting of bacterial isolates, both systems identified 100% of isolates with 100% agreement at the genus level and 96% agreement at the species level. The isolates with disagreement on species level between both systems are listed in Online Resource Table 2. Most of those disagreements (*n* = 19, 82.6%) were within the *E. cloacae* complex. Other disagreements were between *Bacteroides faecis* and *B*. *thetaiotaomicron* (*n* = 1), *Gardnerella leopoldii/swidsinskii* and *G. vaginalis* (*n* = 1), and between *P. hauseri*,* P penneri*, and *P. vulgaris* (*n* = 2).

### Further practical considerations

Further practical considerations are summarized in Table 3.

**Table 3:** Practical aspects considering usability

Property	Biotyper sirius	VITEK MS PRIME
Usability		
Dimensions of device	Tabletop 500 × 1070 × 710 mm (W × H × D)	Tabletop 710 × 1100 × 700 mm (W × H × D)
Weight of device	75 kg	146 kg
Control PC	Separate	On-board PC
Noise emission	< 60 dB	< 65 dB
Ready-to-use matrix solution	No	Yes
Calibration	BTS	*E. coli* ATCC 8739
Ease of target preparation	High	Moderate
Disposable target	Yes	Yes
Reusable target	Yes	No
Capacity per target (spots)	96	48
Loading capacity (spots)	1 × 96	16 × 48
Random access	No	Yes
Database, IVD extensions, and additional kits		
Database	4194 species (BMT IVD reference library 12.0) Mycobacteria and filamentous fungi not included	1316 species (VITEK IVD 3.2 knowledge base) Mycobacteria and filamentous fungi included
IVD software extensions	MBT Mycobacteria IVD Module MBT HT Filamentous Fungi IVD Module MBT Subtyping IVD Module MBT HT Sepsityper IVD Module	No
Additional kits	MBT Sepsityper IVD Kit MBT STAR-Carba IVD Assay MBT STAR-Cepha IVD Assay	VITEK MS Blood Culture Kit (RUO) VITEK MS Mould Kit (IVD) VITEK MS Mycobacteria/Nocardia Kit (IVD)

*W* width, *H* height, *D* depth

Bruker’s matrix solution must be rehydrated and prepared freshly each working day. For the VITEK MS PRIME system, a ready-to-use solution is available. Bruker Daltonics offers both single-use and reusable targets, whereas bioMérieux offers only single-use targets. The Biotyper target has a capacity of 96 spots. For each identification run, one spot must be used for calibration with Bruker’s BTS, which must be prepared from lyophilized material. After an identification run, all open spots are available for further testing. The VITEK MS target holds 48 spots grouped in 3 sections (groups) of 16 spots. For each group of 16 spots, an extra spot for calibration is prepared using *E. coli* ATCC 8739 from a fresh overnight culture that has to be prepared daily. Once a single spot within a group and its calibration spot is used for identification, all unused spots expire and cannot be used for subsequent analysis. The Biotyper sirius can be loaded with one target at a time for MALDI-TOF MS measurement while the “load and go” system of the VITEK MS PRIME can hold up to 16 targets at a time and processes them automatically with the option of prioritizing a distinct target. This feature allows the prioritization of an urgent sample and will automatically proceed to the remaining sequence of tests after the prioritized target is analyzed. Smear preparation on the target slide was handier with the Biotyper targets due to the coating of the surface of the spots making them hydrophilic and slightly abrasive. This resulted in an easy bacterial smear and even distribution of matrix on the spot.

## Discussion

The aim of the study was to compare the two latest MALDI-TOF systems, VITEK MS PRIME (bioMérieux) and the MALDI Biotyper sirius (Bruker Daltonics), regarding workflow performance and provide guidance on the suitability of each system for different types of laboratories. To our knowledge, this is the first study to provide a head-to-head comparison of both systems under routine conditions.

Both systems provide a high identification rate of 97 to 98% for routine isolates despite single-spot measurement. The fraction of high-confidence identifications that a laboratory might choose to accept without further review according to the manufacturers’ IVD workflows was 95% for Biotyper sirius and 93.7% for VITEK MS PRIME, respectively. Previous studies assessing the preceding MALDI-TOF–MS system Biotyper Microflex LT and VITEK MS with the previous databases report comparably high numbers of identification. Martiny et al. report 92.7% identification to the species level for Biotyper Microflex LT and 93.2 for VITEK MS with a set of 986 aerobic bacteria from routine practice [11]. Bilecen et al. report 96.7% and 97.12% identification to the species level for the Biotyper Microflex LT and the VITEK MS, respectively [12]. In a side-by-side comparison, Bardelli et al. found identification rates of 96.7% for the VITEK MS and 97.0% for new VITEK MS PRIME, respectively [13]. One restriction of our study is that only routine isolates were included and that the systems were not challenged with rare bacterial species.

Four (57%) of the unidentified isolates and five (30%) of the low confidence identifications with the Biotyper sirius were *E. coli* and *Klebsiella* isolates. This outcome may be attributed to the mucoid morphology of the colonies as they can lead to thicker smears and generate insufficient identification. CNS constituted the other three (43%) of the unidentified isolates and 10 (59%) of the low confidence identifications with the Biotyper sirius. This finding was also reported for the preceding systems and databases [14, 15]. We did not see these patterns with the VITEK MS PRIME where unidentified isolates and lower confidence scores were distributed more evenly among the species tested.

After retesting previously unidentified isolates with the simple direct transfer method, 100% identification rate was reached with both machines highlighting that the systems are equally suited for the identification of routine bacterial isolates. Furthermore, this result suggests that the major factor for the correct and consistent identification of bacterial isolates is the quality of target preparation. It highly depends on the purity of culture, amount of bacterial biomass smeared on the target and experience of technicians, a finding that has previously been reported for preceding versions [11].

The isolates for which a disagreement on species level was observed mainly belonged to the *Enterobacter cloacae* complex. Correct identification within the *Enterobacter cloacae* complex by MALDI-TOF MS is known to be challenging [16]. For *Proteus hauseri/penneri/vulgaris* and *Bacteroides faecis/thetaiotaomicron*, Bruker’s HT software gives a warning that these species within their genus have very similar mass spectra and are difficult to distinguish, which is the most likely reason for the disagreement between the two systems. As correct and consistent identification of those species is difficult, laboratories might decide to group species together for routine reporting and deduction of intrinsic resistance. In our laboratory, we do so for members of the *Enterobacter cloacae* complex.

The MBT IVD reference library 12.0 includes 4194 species while the VITEK MS IVD 3.2 knowledge base includes 1316 species. For the identification of mycobacteria and filamentous fungi with the Biotyper sirius, extra mycobacteria and Fil Fungi IVD Modules must be purchased. The VITEK MS knowledge base includes those groups.

When deciding on a new MALDI-TOF MS system acquisition and running costs have to be considered. But because prices and reimbursement conditions differ between countries and laboratories, a cost analysis was not performed.

An additional aspect to consider when deciding on a system might be its application in the research field or for routine use only. Both systems can be equipped with research use only (RUO) applications and software. The Biotyper sirius is available in two versions: the Biotyper sirius One, which can operate only in positive ion mode; and the Biotyper sirius, used in this study, which can also be operated in negative ion mode. Negative ion mode allows investigators further research applications like analysis of lipids, for example, determination of colistin resistance [18]. To a certain degree, determination of resistance is also possible from regular spectra obtained in positive ion mode. This was shown for MRSA [19], *bla*_KPC_ detection in *Enterobacterales* [20], and *cfiA*-positive *B. fragilis* strains [21]. The additional MBT Subtyping IVD Module for the Biotyper sirius allows the detection of *bla*_KPC_ in *K. pneumoniae* and *E. coli* and *cfiA*-positive *B. fragilis* strains parallel to the identification procedure with no additional steps.

Furthermore, rapid pathogen identification from positive blood cultures is a major issue in diagnostics of bloodstream infections. For direct identification from positive blood cultures, both companies offer additional kits that are compatible with their machines. Bruker offers an IVD marked solution with the MBT Sepsityper IVD Kit while the VITEK MS Blood Culture Kit is for research use only.

As the analytical performance of both systems for routine bacterial isolates investigated in this study does not differ significantly, practical issues like usability, hands-on time, TAT, TTR, and software integration are likely to determine the choice. The target preparation process for both systems does not differ much. However, the preparation felt easier with the disposable Biotyper targets compared to the disposable VITEK MS slides due to their surface texture and the fact that matrix application can be done in batch within 30 min with the Biotyper IVD workflow whereas the VITEK IVD workflow requires matrix application after each smeared spot. In our study, this is reflected in a reduced hands-on time for the Biotyper target preparation workflow. Biomerieux’s ready-to-use reagents and the *E. coli* ATCC 8739 used for calibration are very convenient to use compared to Bruker’s lyophilized matrix. In our study, we only used disposable targets with both systems. However, the use of reusable steel targets that are only available for the Biotyper system will cut costs at the expense of additional hands-on time for cleaning. Another aspect regarding the usability and effectiveness of target preparation is that VITEK MS’s rigorous calibration format with one calibration spot for a predefined group of 16 spots will likely result in unused spots on the target that will not be available for further testing as the spot for calibration can only be used once. Furthermore, when preparing a VITEK target, the user has to provide the information on whether bacterial or fungal isolate is to be identified. If a bacterial colony is misinterpreted as *Candida* and vice versa by the technicians, the software will not give an identification result but “no ID.” This may result in a higher number of retesting compared to the Biotyper system.

For the measurement and identification process, we saw significant differences between the systems with both having their pros and cons. Notably, both systems improved the TTR for measurement compared to the previous versions of the instruments and software. The preceding Bruker Microflex LT required about 25 to 30 min for the measurement of 48 spots [11, 14, 22]. In comparison, the new Biotyper sirius system, which we evaluated in our study, only took about 6 min for 47 spots. With the preceding VITEK MS system, analyses of 48 spots consumed about 50 min [11, 14, 22]. This time decreased to about 24 min for 47 spots with the new VITEK MS PRIME. In all three scenarios investigated in our study, the TTR was significantly shorter for the Biotyper sirius compared to the VITEK MS Prime. The difference was approximately 39 min for a target with 95 isolates, about 18 min for a target with 47 isolates, and about 30 min for a series of three targets, each spotted with 16 isolates. This saving of time is likely relevant for laboratories with high numbers of identifications, especially during peak hours. With the TTR evaluated for the measurement process in this study, theoretically about 5000 spots could be measured with one Biotyper in an 8-h shift while the VITEK MS Prime can process about one fifth of this. This theoretical capacity of the Biotyper is hampered by the fact that the system has no random access for target loading. This function is provided with the VITEK load-and-go system that holds up to 16 targets and significantly reduces hands-on time by about 1.5 to 2 min per target. This relatively small difference in hands-on time might not be important for laboratories that prepare few targets. However, for laboratories that handle many targets, this can become a relevant factor. This makes the VITEK MS PRIME a reasonable choice for laboratories with overall fewer numbers of identifications, but higher amount of targets and a workflow designed for parallel processing on different workstations. Due to its high speed of measurement and spectra interpretation, the Biotyper sirius system is suited for laboratories that have high numbers of identifications and a workflow designed for processing larger batches.

Overall, both systems exhibit good performance and have their strengths and weaknesses. With this study, we tried to provide some guidance for CMLs that face the choice of a new MALDI-TOF MS system tailored to their individual criteria, needs, and preferences.

### Supplementary Information

ESM 1 (PDF 784 KB)

## Data Availability

The datasets generated during and/or analyzed during the current study are available from the corresponding author on reasonable request.
