# Supplementary material for: A side-by-side comparison of the new VITEK MS PRIME and the MALDI Biotyper sirius in the clinical microbiology laboratory
Source: Eur J Clin Microbiol Infect Dis. 2023 Oct 5;42(11):1355–63. doi: 10.1007/s10096-023-04666-x (PMC10587274; doi:10.1007/s10096-023-04666-x)
Supplement: Supplementary file 1 — (PDF 784 KB) [file 10096_2023_4666_MOESM1_ESM.pdf]

A side-by-side comparison of the new VITEK® MS PRIME and the Bruker Biotyper® sirius MALDI-TOF MS systems in the clinical microbiology laboratory

Philipp Thelen, Sandra Gräber, Erika Schmidt and Axel Hamprecht

Supplementary Material

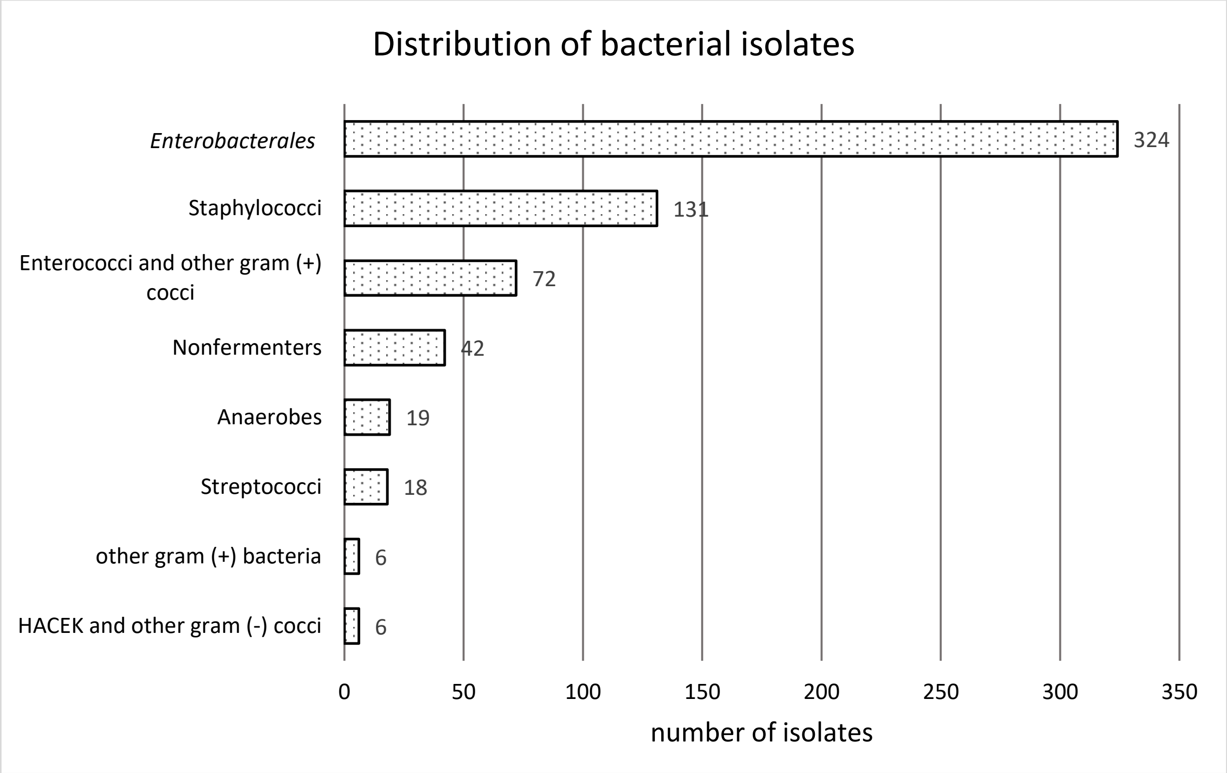

Fig. 1 species distribution of bacterial isolates

TABLE 1 Summary of MALDI-TOF MS results for 618 isolates

			Biotyper® sirius				VITEK® MS PRIME			
**Genus and species**	No. of isolates	% isolates	log(score) >= 2	low consistency	log(score) 1.7 - 1.99	No identification	Confidence score 99.9 %	Confidence score 60-99.8 %	low discrmination	No identification
					**Enterobacterales (20 species)**					
Escherichia coli	172	27.83%	165	0	4	3	168	2	1	1
Citrobacter amalonaticus	2	0.32%	2	0	0	0	2	0	0	0
Citrobacter braakii	1	0.16%	0	1	0	0	0	1	0	0
Citrobacter freundii	4	0.65%	4	0	0	0	4	0	0	0
Citrobacter koseri	9	1.46%	9	0	0	0	9	0	0	0
Enterobacter cloacae complex	22	3.56%	21	1	0	0	0	0	21	1
Klebsiella aerogenes	2	0.32%	2	0	0	0	2	0	0	0
Klebsiella oxytoca	9	1.46%	9	0	0	0	8	1	0	0
Klebsiella pneumoniae	43	6.96%	41	0	1	1	38	1	0	4
Klebsiella variicola	1	0.16%	1	0	0	0	0	0	1	0
Proteus mirabilis	33	5.34%	33	0	0	0	33	0	0	0
Proteus hauseri / penneri / vulgaris	0	1.46%	8	1	0	0	8	0	1	0
Serratia liquefaciens	1	0.16%	1	0	0	0	1	0	0	0
Serratia marcescens	4	0.65%	4	0	0	0	4	0	0	0
Serratia proteamaculans	1	0.16%	1	0	0	0	1	0	0	0
Morganella morganii	5	0.81%	5	0	0	0	5	0	0	0
Lelliottia amnigena	1	0.16%	1	0	0	0	0	0	0	1
Providencia stuartii	3	0.49%	3	0	0	0	3	0	0	0
Raoultella ornithinolytica	1	0.16%	1	0	0	0	1	0	0	0
Salmonella species	1	0.16%	1	0	0	0	1	0	0	0

(Continued on next page)

TABLE 2 (Continued)

			Biotyper® sirius				VITEK® MS PRIME			
**Genus and species**	No. of isolates	% isolates	log(score) >= 2	low consistency	log(score) 1.7 - 1.99	No identification	Confidence score 99.9 %	Confidence score 60-99.8 %	low discrmination	No identification
**Nonfermenters (7 species)**										
Pseudomonas aeruginosa	30	4.85%	30	0	0	0	29	1	0	0
Stenotrophomonas maltophilia	6	0.97%	6	0	0	0	6	0	0	0
Acinetobacter baumannii	1	0.16%	1	0	0	0	1	0	0	0
Acinetobacter baylyi	1	0.16%	1	0	0	0	0	0	0	1
Acinetobacter pittii	1	0.16%	1	0	0	0	1	0	0	0
Acinetobacter ursingii	1	0.16%	1	0	0	0	1	0	0	0
Delftia acidovorans	2	0.32%	0	2	0	0	2	0	0	0
**Enterococci and other gram (+) cocci (3 species)**										
Enterococcus faecalis	44	7.12%	43	0	1	0	43	1	0	0
Enterococcus faecium	27	4.37%	27	0	0	0	26	0	0	1
Aerococcus urinae	1	0.16%	1	0	0	0	1	0	0	0
**Staphylococci (10 species)**										
Staphylococcus aureus	72	11.65%	72	0	0	0	72	0	0	0
Staphylococcus capitis	1	0.16%	1	0	0	0	1	0	0	0
Staphylococcus caprae	2	0.32%	1	0	1	0	2	0	0	0
Staphylococcus epidermidis	39	6.31%	33	0	5	1	39	0	0	0
Staphylococcus haemolyticus	14	2.27%	9	0	3	2	14	0	0	0
Staphylococcus hominis	1	0.16%	1	0	0	0	1	0	0	0
Staphylococcus lugdunensis	1	0.16%	0	0	1	0	1	0	0	0
Staphylococcus saprophyticus	1	0.16%	1	0	0	0	1	0	0	0
Staphylococcus saccharolyticus	1	0.16%	1	0	0	0	1	0	0	0

(Continued on next page)

TABLE 3 (Continued)

			Biotyper® sirius				VITEK® MS PRIME			
**Genus and species**	No. of isolates	% isolates	log(score) >= 2	low consistency	log(score) 1.7 - 1.99	No identification	Confidence score 99.9 %	Confidence score 60-99.8 %	low discrmination	No identification
					**Streptococci (7 species)**					
Streptococcus agalactiae	8	1.29%	8	0	0	0	6	2	0	0
Streptococcus constellatus	1	0.16%	1	0	0	0	1	0	0	0
Streptococcus dysgalactiae	1	0.16%	1	0	0	0	0	0	1	0
Streptococcus infantarius	1	0.16%	0	1	0	0	1	0	0	0
Streptococcus mitis_oralis	5	0.81%	5	0	0	0	3	1	0	1
Streptococcus sanguinis	1	0.16%	1	0	0	0	1	0	0	0
Streptococcus vestibularis	1	0.16%	0	1	0	0	0	0	1	0
					**HACEK and other gram (-) cocci (6 species)**					
Haemophilus influenzae	1	0.16%	1	0	0	0	1	0	0	0
Haemophilus parainfluenzae	1	0.16%	1	0	0	0	1	0	0	0
Kingella kingae	1	0.16%	1	0	0	0	1	0	0	0
Neisseria flavescens/subflava group	1	0.16%	1	0	0	0	1	0	0	0
Paracoccus yeei	1	0.16%	1	0	0	0	1	0	0	0
Roseomonas mucosa	1	0.16%	1	0	0	0	1	0	0	0
					**other gram (+) bacteria (5 species)**					
Bacillus cereus	1	0.16%	1	0	0	0	0	1	0	0
Corynebacterium striatum	2	0.32%	2	0	0	0	2	0	0	0
Gardnerella leopoldii / swidsinski	1	0.16%	1	0	0	0	0	0	0	1
Gardnerella vaginalis	1	0.16%	1	0	0	0	1	0	0	0
Rothia mucilaginosa	1	0.16%	1	0	0	0	1	0	0	0

(Continued on next page)

TABLE 4 (Continued)

			Biotyper® sirius				VITEK® MS PRIME			
**Genus and species**	No. of isolates	% isolates	log(score) >= 2	low consistency	log(score) 1.7 - 1.99	No identification	Confidence score 99.9 %	Confidence score 60-99.8 %	low discrmination	No identification
					**Anaerobes (13 species)**					
Bacteroides fragilis	4	0.65%	4	0	0	0	4	0	0	0
Bacteroides faecis / thetaiotaomicron	2	0.32%	2	0	0	0	2	0	0	0
Clostridium perfringens	1	0.16%	1	0	0	0	1	0	0	0
Clostridium ramosum	1	0.16%	1	0	0	0	1	0	0	0
Finegoldia magna	1	0.16%	0	0	1	0	1	0	0	0
Lactobacillus crispatus	1	0.16%	1	0	0	0	1	0	0	0
Lactobacillus jensenii	1	0.16%	1	0	0	0	1	0	0	0
Parvimonas micra	1	0.16%	1	0	0	0	1	0	0	0
Phocaeicola vulgatus	1	0.16%	1	0	0	0	1	0	0	0
Prevotella buccae	1	0.16%	1	0	0	0	1	0	0	0
Prevotella veroralis	1	0.16%	1	0	0	0	1	0	0	0
Schaalia turicensis	1	0.16%	1	0	0	0	0	0	0	1
Schaalia odontolytica	2	0.32%	2	0	0	0	1	0	0	1
**Total**	**618**		**587 (95.0%)**	**7 (1.1%)**	**17 (2.7%)**	**7 (1.1%)**	**568 (91.9%)**	**11 (1.8 %)**	**26 (4.2) %**	**13 (2.1%)**

Table 2 Identifications with disagreement on species level on both systems. Species with a slash indicate that the software was not able to reliably distinguish between both species

	Biotyper® sirius	VITEK® MS PRIME	N (%)
Species identification	*Enterobacter hormaechei*	*Enterobacter asburiae/cloacae*	12 (52.2)
	Enterobacter bugandensis	*Enterobacter asburiae/cloacae*	3 (13.0)
	Enterobacter kobei	*Enterobacter asburiae/cloacae*	2 (8.7)
	Enterobacter ludwigii	*Enterobacter asburiae/cloacae*	2 (8.7)
	Bacteroides faecis	*Bacteroides thetaiotaomicron*	1 (4.3)
	Gardnerella leopoldii/swidsinskii	*Gardnerella vaginalis*	1 (4.3)
	Proteus hauseri/vulgaris	*Proteus penneri*	1 (4.3)
	Proteus hauseri	*Proteus vulgaris*	1 (4.3)
